# Adsorption Free Energy of Cellulose Nanocrystal on Water–Oil Interface

**DOI:** 10.3390/nano12081321

**Published:** 2022-04-12

**Authors:** Kenya Ito, Mitsuhiro Matsumoto

**Affiliations:** Graduate School of Engineering, Kyoto University, Kyoto 615-8540, Japan; ito.kenya.77e@st.kyoto-u.ac.jp

**Keywords:** cellulose nanocrystal, amphiphilicity, adsorption free energy, potential of mean force, confined liquid, molecular dynamics simulation

## Abstract

To investigate the amphiphilicity of cellulose, a series of molecular dynamics simulations were performed with a cellulose nanocrystal and a water–octane interfacial system. Assuming that the axis of cellulose is parallel to the water–octane interface, the freedoms of motion of the nanocrystal were restricted to two, the distance from the interface and the orientation around the axis. The mean force and the mean torque on the nanocrystal were evaluated with sufficiently long simulation at each crystal configuration, and their numerical integration gave a smooth free energy surface as the potential of mean force. The cellulose sample used here was found to be much more hydrophilic than oleophilic with the free energy difference $\Delta F_{w\to o}=318$ kcal/mol. Three adsorption states with local minimum of adsorption free energy are distinguished in the free energy surface—the direct contact type which is similar to previously reported one, the hydrophilic-surface/water/octane type where a thin water layer is sandwiched between the surface and the octane phase, and the oleophilic/water/octane type where a thin water layer also exists. Water molecules in these water layers contribute to stabilize the adsorption states by taking a special orientational order and slow self-diffusion.

## 1. Introduction

Cellulose is one of the abundant sustainable materials and has been used in various forms, from conventional papers and textiles to more advanced novel materials for films, composites, coatings, and emulsifiers [1,2,3,4]. In this paper, we focus on its amphiphilicity, which is the most important property in its application as emulsifier [2].

**Figure 1:**
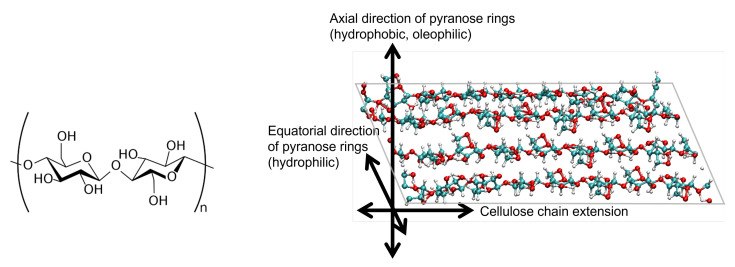
(**Left**) Unit of cellulose chain; (**Right**) Typical atomic configuration of a cellulose sheet showing characteristic directions. Cyan: carbon atoms, red: oxygen, white: hydrogen.

The origin of cellulose amphiphilicity exists in its molecular structure. A single molecular chain consists of β 1–4 linked D-glucose units. Each unit (β-D-glucose) has a six-atom ring (five carbon atoms with one oxygen, i.e., a pyranose ring) with two hydroxy groups and one -CH_2_OH group attached to the ring carbons. The hydroxy groups easily make hydrogen bonds between cellulose chains, leading to a sheet, as shown in Figure 1; these sheets stack to form a nanocrystal. Thus, such nanocrystals are typically hydrophobic or oleophilic on surface normal to (i.e., toward the axial directions of) the pyranose rings, while being hydrophilic on surface toward the equatorial directions. The amphiphilicity thus originates from this anisotropic property of crystalline structure of cellulose at atomic scales.

This amphiphilicity of cellulose is utilized to stabilize various types of emulsions; a relevant review is found in [2]. After the pioneering work by Oza and Frank [5], a number of experimental investigations have been conducted on emulsification with cellulose nanofibers and nanocrystals, such as using cellulose hydrogels [6] or amorphous [7], investigating the aspect ratio (ratio of length to width of the cellulose nanocrystal) effect [8], X-ray structural analysis and thermal stability investigation [9], and structural analysis with small-angle neutron scattering [10]. In general, these experimental investigations indicate that cellulose nanofibers and nanocrystals work as good stabilizers of oil-in-water emulsions due to their amphiphilicity; however it is often reported that cellulose in its pristine states is more hydrophilic than oleophilic, which brings difficulty in realizing water-in-oil-type emulsions. It would be beneficial to control its amphiphilicity—more detailed understanding of the hydrophilicity and oleophilicity of cellulose is thus required.

In atomic scales, several studies with molecular dynamics (MD) simulations have been reported regarding the amphiphilicity of cellulose. Biermann et al. investigated the hydrophilic property by evaluating the local chemical potential of water molecules near a cellulose crystal [11]. Alqus et al. traced in some detail the dynamics of chains in a cellulose crystal and water molecules near a graphene sheet, suggesting the mechanism of cellulose stabilization on the graphene [12]. More recently, Miyamoto et al. directly investigated the dynamic process of emulsification with cellulose nanocrystal in water–octane mixture [13], clearly indicating that a stable state of adsorption on an oil droplet was formed after a long simulation of 80 ns. This is the first atomic-level investigation of the emulsification process, giving much insight into the adsorption behavior of cellulose nanocrystals [2], although the size of “emulsion” (about 4 nm in radius) is much smaller than typical experimental systems due to the computational limitation.

Since the adsorption of cellulose is a fairly slow process including mass diffusion, detailed understanding of dynamic behavior during such an emulsification process is not sufficient yet. In this paper, we propose another theoretical approach, i.e., investigation of the thermodynamics by evaluating the adsorption free energy with a series of molecular simulations in a wide configurational range of cellulose nanocrystal.

Determining the free energy surface in specified parametric spaces is an important topic in various fields of chemical physics, and a large number of methods with molecular simulation techniques have been proposed, such as evaluating the potential of mean force by weighted samplings for biopolymers [14], metadynamics samplings in a reaction coordinate space [15], and path samplings in a molecular configurational space [16]. A recent report on quantum MD simulations to determine the reaction paths in surface deposition processes [17] can belong to the category of path samplings. Here we adopt a simple approach to evaluate the potential of mean force based on the “reversible work theorem” [18] combined with classical MD simulations.

## 2. Models and Simulation Methods

We adopted the software package LAMMPS [19] to perform all simulations with the MD method.

### 2.1. Cellulose Nanocrystal Model

**Figure 2:**
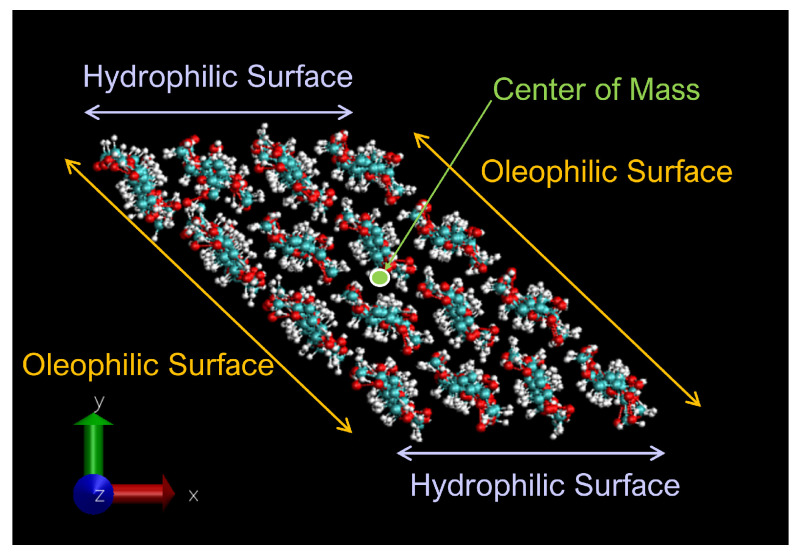
Cellulose nanocrystal consisting of 4×4 cellulose chains; cross-sectional view. The orientational angle $\theta_{z}=0$ for this configuration.

The cellulose nanocrystal used in the simulation consists of 4×4 cellulose chains, as shown in Figure 2. Each chain has eight units of D-glucose, which are β-linked along the *z* direction. We impose the periodic boundary condition along the chain (the *z* direction in our system) so that each chain is expected to be infinitely long.

For the MD simulations, we adopt the CHARMM general force field (CGenFF) [20] for atomic interactions, which consists of partial electric charge on each atom, Lennard-Jones (LJ) interactions between non-bonded atoms, harmonic potentials for chemical bonds, angular and torsional potentials. After manually setting up the initial atomic configuration based on the crystallographic data of cellulose II [21], we energetically optimized it in vacuum. The optimized crystal takes a parallelepiped shape, with two oleophilic surfaces which are normal (i.e., axial direction) to pyranose rings, and two hydrophilic ones which are equatorial to the rings, as shown in Figure 2. The area ratio of this sample, oleophil:hydrophil, is about 1.8:1. The nanocrystal was then further equilibrated in vacuum with temperature maintained at T=300 K for 1 ns; the crystal structure is stable enough to be maintained during the equilibration. This structure corresponds to cellulose II; the stability of this structure agrees with a report by Kroon-Batenburg et al. [22], in which the structural stability of several cellulose crystals is compared at T=300 K with MD simulations.

### 2.2. Simulation System

A rectangular simulation cell with periodic boundary conditions for all directions is prepared, half of which is filled with water and the other half with oil. Liquid *n*-octane (C_8_H_18_) is used as the oil, similar to the setup in [13]. The TIP3P model [23] is adopted for water molecules. The CGenFF model is used for octane molecules. The conventional Lorentz–Berthelot rule is assumed for the LJ interactions between non-bonded atoms. Preliminary MD simulation yields the interfacial tension of 58.0 mN/m at T=300 K, which fairly agrees with reported values 61.5 mN/m at 293 K with molecular simulations [24] and 48.9 mN/m at 25 °C with experiments [25].

**Figure 3:**
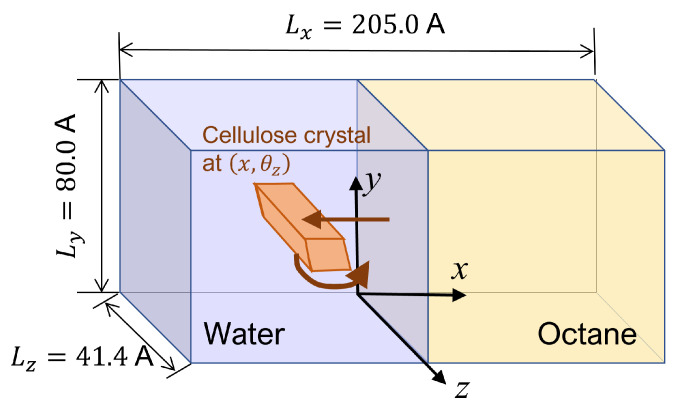
Schematic view of the simulation system.

After equilibrating this interfacial system, a cellulose nanocrystal is inserted with the cellulose axis parallel to the *z* direction, as shown in Figure 3. Since the cellulose chains are “infinitely” long due to the periodic boundary condition along *y*, and the water–oil interface is essentially flat, the problem of extremely large curvature in the MD simulation of emulsification [13] is overcome in our setup. Considering the symmetry of configuration, we can reduce the number of degrees of freedom for the cellulose nanocrystal to two, i.e., the distance of the nanocrystal from the water–oil interface and its orientation. Here, we choose the two as *x* which is the position of the center-of-mass axis (along *z* direction) and $\theta_{z}$ which is the rotational angle around the axis, as shown in Figure 3. The coordination origin of *x* is placed on the water–oil interface, while $\theta_{z}$ is defined to be zero when the hydrophilic surface of the crystal is perpendicular to the interface (*y*–*z* plane). Note that only the position *x* and the angle $\theta_{z}$ are fixed during all simulations, leaving other internal degrees of freedom of the crystal intact. The crystal was still stable enough at any condition $\left(x,\theta_{z}\right)$.

The natural “length” of the cellulose chain in the nanocrystal used here is 41.4 Å, which determines the cell size along the *z* direction. The “width” of the hydrophilic surface is about 20 Å while that of the oleophilic one is about 36 Å. Therefore, we need sufficiently large sizes of water and oil phases. We chose 205 Å as the cell size along the *x* direction and 80 Å along the *y* direction.

### 2.3. MD Simulation

Since most of experiments for emulsifier application were conducted at room temperature, all simulations were carried out under canonical ensemble conditions with controlled temperature at T=300 K, using the Nosé–Hoover thermostat algorithm [26]. The PPPM method [27] was utilized to treat the long-range Coulombic interactions.

Detailed conditions for the simulation are summarized in Table 1.

### 2.4. Construction of Free Energy Surface

Owing to the symmetry of the system, we conceive that the Helmholtz free energy *F* of the system depends only on the position *x* and the rotation angle $\theta_{z}$. The *x* component of the force $f_{x}$ and the *z* component of the torque $T_{z}$ exerted on the nanocrystal should be obtained from its partial derivative as

(1) $$f_{x}=-{\left(\frac{\partial F}{\partial x}\right)}_{\theta_{z}}$$

and

(2) $$T_{z}=-{\left(\frac{\partial F}{\partial \theta_{z}}\right)}_{x}$$

Thus, we can evaluate the adsorption free energy $F(x,\theta_{z})$, or the potential of mean force, by integrating their time average [14,18] as

(3) $$F\left(x,\theta_{z}\right)-F\left(x_{0},\theta_{z}\right)=-\int_{x_{0}}^{x}\langle f_{x}\left(x^{\prime },\theta_{z}\right)\rangle dx^{\prime }$$

and

(4) $$F\left(x,\theta_{z}\right)-F\left(x,\theta_{z0}\right)=-\int_{\theta_{z0}}^{\theta_{z}}\langle T_{z}\left(x,\theta_{z}^{\prime }\right)\rangle d\theta_{z}^{\prime }$$

As the reference state, we have chosen $x_{0}=-30$ Å; we confirmed that $\langle T_{z}\left(x=\pm 30\;Å,\theta_{z}\right)\rangle$ is essentially zero, which suggests that the effects of water/oil interface on the cellulose nanocrystal are negligibly small when the cellulose is placed by 30 Å far from the interface.

To perform these numerical integrations, we discretized the parameter space $\left(x,\theta_{z}\right)$ with constant Δx and $\Delta \theta_{z}$. Two types of grid were used:

- First, we adopted a coarse grid to roughly investigate the free energy behavior: −30≤x[Å]≤+30 with Δx=5 Å and $0\leq \theta_{z}\;{[}^{\circ }]<180$ with $\Delta \theta_{z}=15^{\circ }$. The number of grid points is thus 13×12;
- To evaluate the local minima of the free energy surface more closely, a finer grid was adopted: −25≤x[Å]≤−10 with Δx=1 Å and $75\leq \theta_{z}\;{[}^{\circ }]\leq 175$ with $\Delta \theta_{z}=5^{\circ }$. The number of grid points on this finer grid is 16×16.

The procedure to obtain the free energy surface based on the data $\langle f_{x}\rangle$ and $\langle T_{z}\rangle$ on the coarse grid points is as follows.

Step 1: Define $F(-30\;Å,\theta_{z})=0$ irrespective of $\theta_{z}$, which is the “origin” of the free energy.
Step 2: For each $\theta_{z}$, integration (or summation) in Equation (3) is carried out to obtain preliminary values of $F(+30\;Å,\theta_{z})$. $F(+30\;Å,\theta_{z})$ is expected to be constant, but a small $\theta_{z}$ dependence remains due to the statistical fluctuations.
Step 3: Assume the average value ${\langle F(+30\;Å,\theta_{z})\rangle }_{\theta_{z}}$ as the free energy difference $\Delta F_{w\to o}$, and give it to $F(+30\;Å,\theta_{z})$ for all $\theta_{z}$. Thus the boundary values of $F(\pm 30\;Å,\theta_{z})$ are determined at this step.
Step 4: Choose a single grid point $\left(x,\theta_{z}\right)$, x≠±30 Å, randomly.
Step 5: Refine the data at the point using the four neighbor points. With the notation $F_{i,j}$ for $F(x,\theta_{z})$, $f_{i,j}$ for the force component $f_{x}$, and $T_{i,j}$ for the torque component $T_{z}$ on grid point (i,j), the update is performed as
  (5) $$\begin{array}{ccc} 4F_{i,j} & \leftarrow & \left(F_{i-1,j}-\frac{1}{2}\left[f_{i-1,j}+f_{i,j}\right]\Delta x\right)+\left(F_{i+1,j}+\frac{1}{2}\left[f_{i,j}+f_{i+1,j}\right]\Delta x\right)\\ & & \left(F_{i,j-1}-\frac{1}{2}\left[T_{i,j-1}+T_{i,j}\right]\Delta \theta_{z}\right)+\left(F_{i,j+1}+\frac{1}{2}\left[T_{i,j}+T_{i,j+1}\right]\Delta \theta_{z}\right)\\ & = & F_{i-1,j}+F_{i+1,j}+F_{i,j-1}+F_{i,j+1}\\ & & +\frac{1}{2}\left[-f_{i-1,j}+f_{i+1,j}\right]\Delta x+\frac{1}{2}\left[-T_{i,j-1}+T_{i,j+1}\right]\Delta \theta_{z} \end{array}$$
Step 6: Repeat Steps 4 and 5 enough times to reach a steady state. For our system, a smooth surface of $\Delta F(x,\theta_{z})$ was obtained after $10^{6}$ repeats.

A similar procedure is adopted for the finer grid, except that the boundary values of *F* are fixed to the previously obtained results of the coarse grid calculation.

## 3. Results and Discussion

### 3.1. Sampling

**Figure 4:**
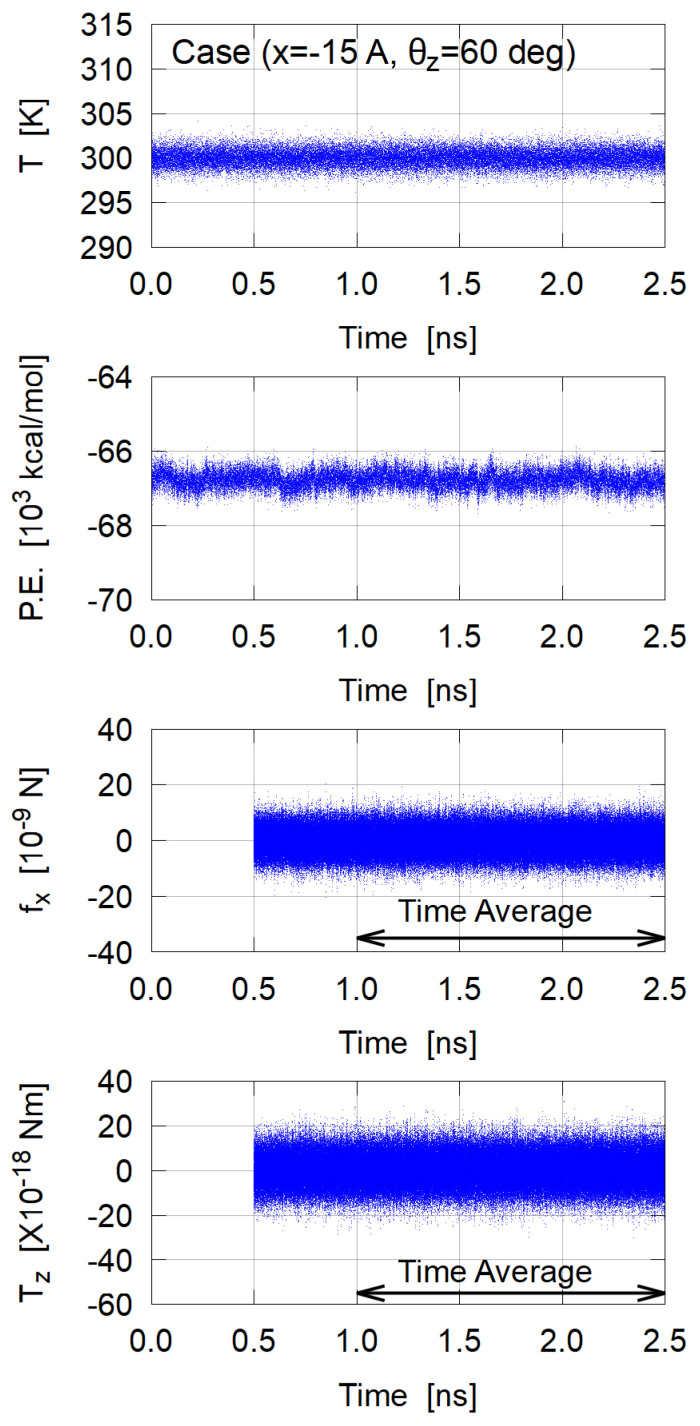
Variations of temperature *T*, potential energy (P.E.), force component $f_{x}$, and torque component $T_{z}$; an example of $\left(x=-15\;Å,\theta_{z}=60^{\circ }\right)$ case. In this case, $\langle f_{x}\rangle =-0.2068\times 10^{-9}$ N and $\langle T_{z}\rangle =0.3921\times 10^{-18}$ Nm were obtained as the time average over 1.5 ns.

An example of data fluctuations during each MD simulation is shown in Figure 4. Since the system seems to reach an equilibrium state after 1.0 ns, we calculated the time average of $f_{x}$ and $T_{z}$ from 1 ns to 2.5 ns for each case. Both $f_{x}$ and $T_{z}$ largely fluctuate around zero, but $3\times 10^{5}$ sampling data for 1.5 ns seem sufficient to obtain a reasonable time average.

### 3.2. Free Energy Surface

#### 3.2.1. Global View

**Figure 5:**
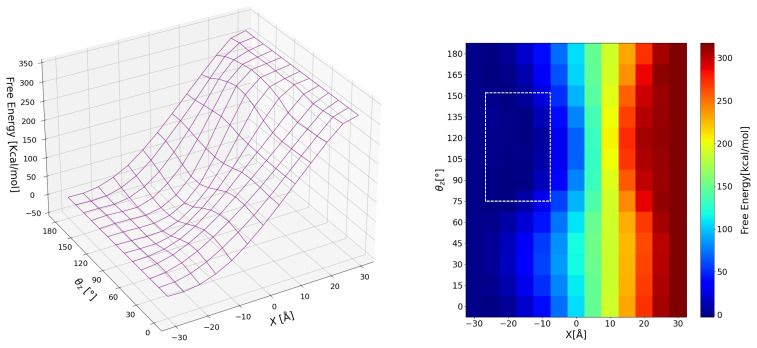
Adsorption free energy; (**Left**) free energy surface, (**Right**) contour color map. The area indicated with dashed lines in the color map is the parameter range for investigation with the finer grid.

The free energy surface in the whole parameter space obtained with the coarse grid (Δx=5 Å and $\Delta \theta_{z}=15^{\circ }$) is shown in Figure 5 (Left) and the contour color map in Figure 5 (Right). Contrary to our expectation of cellulose amphiphilicity, this nanocrystal sample is much more hydrophilic than oleophilic; the free energy difference between the sample in “bulk” water and that in “bulk” octane, $\Delta F_{w\to o}$, is as much as 318 kcal/mol, suggesting that the cellulose crystal is much stabilized in water due to its solvation (hydration). This strong hydrophilicity of pristine (unmodified) cellulose nanocrystal, which restricts the application as emulsifiers, has often been mentioned [8,28]. In principle, the free energy difference $\Delta F_{w\to o}$ is an observable thermodynamic quantity in experiments, but no values to be compared are found to the best of our knowledge. The large value of $\Delta F_{w\to o}≃318$ kcal/mol suggests that this nanocrystal is essentially insoluble in oil, which certainly brings difficulty in thermodynamic measurements.

#### 3.2.2. Detailed View

**Figure 6:**
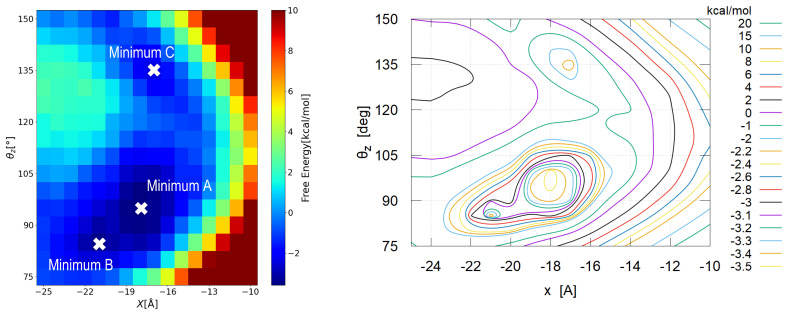
Adsorption free energy on finer grid; (**Left**) contour color map indicating three local minima, (**Right**) contour line map for the same data. The contour lines are drawn with eighth-order B-spline interpolations.

Having noticed that there are several local minima in Figure 5, we took a closer look at the free energy surface with the finer grid. The results are shown in Figure 6 (Left) as a contour color map, which suggests three local minima in this parameter range. The contour line map shown in Figure 6 (Right) indicates these minima more clearly. In the following discussion, we name them Type A, Type B and Type C from the deepest.

Type A and Type B are very close in the parameter space $\left(x,\theta_{z}\right)$ with a low energy barrier of about 0.2 kcal/mol between them. Thus we expect that, once the adsorbed nanocrystal takes the Type B state, it easily reaches the most stable state, Type A.

The situation of Type C seems a little different. The barrier height between Type C and Type A is about 2.5 kcal/mol (∼1300 K in temperature unit); once the nanocrystal takes the Type C configuration, it becomes fairly stable.

### 3.3. Typical Adsorption States

Examples of snapshot at each adsorption state are indicated in Table 2 with the value of adsorption free energy ΔF, which is defined as

(6) $$\Delta F\left(x,\theta_{z}\right)\equiv F\left(x,\theta_{z}\right)-F\left(x=-30\;Å\right).$$

Note that we assume no $\theta_{z}$ dependence of *F* at x=−30 Å (in the “bulk” water phase).

In the most stable state, Type A, the nanocrystal contacts the oil phase on a crystal edge. The adsorption free energy is ΔF≃−3.6 kcal/mol (≃1800 K), suggesting that the nanocrystal sufficiently stabilizes the water–oil interface by adsorption. The orientation of Type A looks similar to that found in [13], where a stable state with direct contact on an edge was reached after 80 ns simulation (Figure 7 (f’) of Ref. [13]).

Two other stable states exist in the vicinity of Type A configuration. In the Type B configuration, the orientation $\theta_{z}$ is close to that of Type A, but the position *x* is slightly apart from the water–oil interface. The snapshot shown in Figure 2 indicates that a thin water layer exists between the hydrophilic surface of the nanocrystal and the oil phase; the typical thickness of the water layer is 3–6 Å. More interesting is the third state Type C, where a thin water layer also exists between the oleophilic surface and the oil phase; this water layer seems to hinder the direct contact of the oleophilic surface with the oil.

We had expected that the nanocrystal would be adsorbed on the water–octane interface simply with its oleophilic surface directly contacted with octane phase, but the obtained free energy surface suggests more complicated behaviors; due to the strong hydrophilicity, the nanocrystal is always covered by water layers even when approaching the interface. To the best of our knowledge, no direct experimental supports for this picture exist; no information on crystal surface is available in SEM images [8,10]; it would be interesting if an observation of nanocrystal with largely different geometry (ratio of oleophilic to hydrophilic surface) were conducted.

We suppose that the thin water layer observed in Type B and Type C may have some special characters to stabilize these configurations. Thus, in the following subsections, static and dynamic properties of water molecules in the gap are investigated.

### 3.4. Orientation of Water Molecules

It is somewhat strange that a stable thin water layer exists between the nanocrystal surface and the oil phase in the stable states, in particular for the Type C adsorption. We suppose that water molecules with some special configuration in the layer may contribute to stabilize the adsorption states.

**Figure 7:**
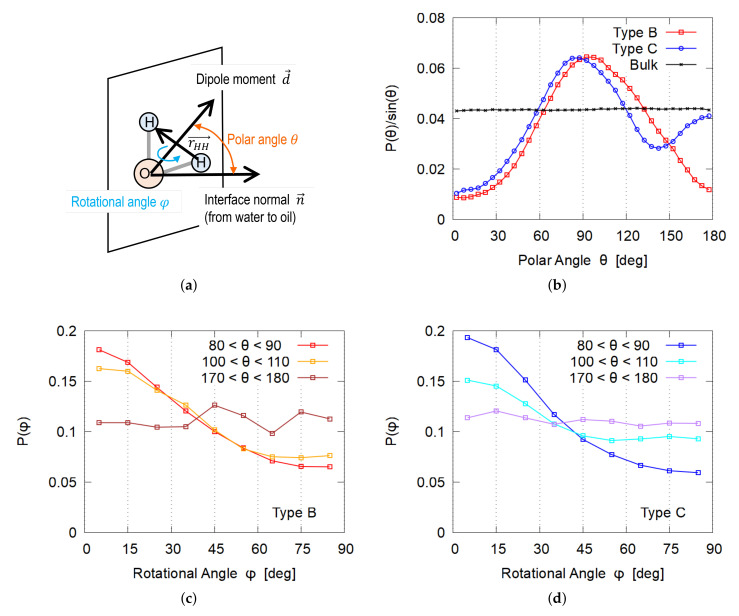
Orientational distribution of water molecules in thin water layer between the adsorbed cellulose nanocrystal and the oil phase. “Bulk” in (**b**) indicates the orientation distribution of water molecules far from both the cellulose and the water–oil interface. (**a**) Definition of orientational angles, θ and φ. (**b**) Distribution of θ. (**c**) Distribution of φ for Type B. (**d**) Distribution of φ for Type C.

First, we investigate the orientation of water molecules in the gap between the nanocrystal and the oil. Here, two orientational angles are defined (Figure 7a). The polar angle θ between the dipole moment $\vec{d}$ of each water molecule and the surface normal $\vec{n}$, where $\vec{n}$ is defined to direct from the water phase to the oil, or $\vec{n}$ is parallel to the *x* axis. The rotational angle φ around $\vec{d}$ is defined as the angle between ${\vec{r}}_{\mathrm{HH}}$ (vector from one hydrogen atom to another) and $\vec{d}\times \vec{n}$, thus, φ=0 means that the ${\vec{r}}_{\mathrm{HH}}$ is parallel to the water–oil interface. The distribution of θ is shown in Figure 7b, where the probability P(θ) with the Jacobian weight correction sinθ is plotted. Plotted in Figure 7c,d present the distribution of φ for specified θ.

Most of the water molecules in the gap of Type B (i.e., between the hydrophilic surface of nanocrystal and the oil phase) tend to lie on the crystal surface because the distribution has a peak at $\theta =90^{\circ }$ (Figure 7b) and $\varphi =0^{\circ }$ (Figure 7c). Detailed inspection of θ distribution reveals that dipole moments have a tendency to direct to the nanocrystal, i.e., the peak in P(θ) slightly shifts to larger values than $90^{\circ }$. This orientational preference is similar to that in a water–vacuum interface [29,30] and water–organic molecular liquid interface [31,32].

The general preference is similar for Type C adsorption, but a slight difference exists; P(θ) has another peak at $\theta ≃180^{\circ }$ (Figure 7b), which corresponds to water molecules with their dipole moment toward the nanocrystal. Closer inspection reveals that the oleophilic surface of the nanocrystal has some “roughness” and water molecules adsorbed in grooves between the molecular chains of cellulose tend to take this exceptional orientation. The number of water molecules in the groove was found to be about one-quarter of all molecules in the gap.

### 3.5. Diffusion of Water Molecules

To further investigate the stability of the water layer between the cellulose and the oil phase, the self-diffusion of water molecules in the layer is evaluated. The mean square displacement (MSD) is defined along each direction as

(7) $${\mathrm{MSD}}_{\alpha }\left(t\right)\equiv {\langle {\left[\alpha_{i}\left(t_{0}+t\right)-\alpha_{i}\left(t_{0}\right)\right]}^{2}\rangle }_{i,t_{0}}\;\left(\alpha =x,y,z\right),$$

where $\alpha_{i}\left(t\right)$ is the position of water molecule *i* inside the gap at time *t*, and 〈〉 indicates the average over time $t_{0}$ and molecule *i*. Sampling was performed with position data for 0.5 ns. The number of water molecules for the sampling (i.e., the molecules which exist in the gap during the sampling period) is 101 for Type B and 148 for Type C, respectively. The diffusion coefficient $D_{\alpha }$ for each direction is evaluated as half of the slope of ${\mathrm{MSD}}_{\alpha }\left(t\right)$ at sufficiently large *t*.

**Figure 8:**
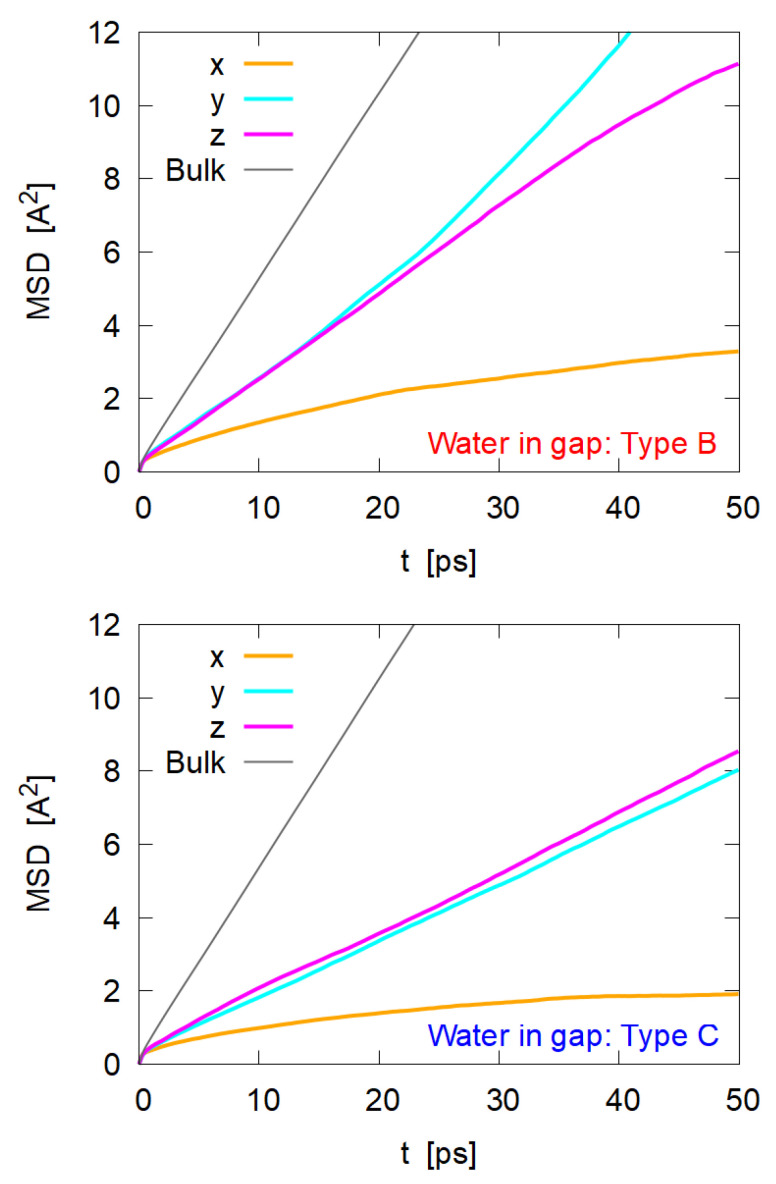
Mean square displacement (MSD) of water molecules in the gap region between the adsorbed cellulose nanocrystal and the oil phase. “Bulk” indicates the MSD of water molecules far from both the cellulose and the water–oil interface.

The obtained ${\mathrm{MSD}}_{\alpha }\left(t\right)$ are shown in Figure 8, which indicates that the motion of water molecules in the gap region are much affected by the cellulose nanocrystal. The following points seem relevant:

- Since the gap distance is only 3–6 Å, it is reasonable that the motion along the *x* direction would be highly restricted;
- Motions along the *y* and *z* directions (i.e., parallel to the nanocrystal surface) are also restricted, but they still show 1/3 to 1/2 mobility of molecules in bulk phase;
- Comparing Type B and Type C, the diffusion in Type B is faster than that in Type C, suggesting that water between the oleophilic surface and the oil phase (Type C) is more structured or ordered;
- For Type B, water molecules seem to diffuse more easily to the *y* direction (i.e., crossing cellulose chains) than to the *z* direction along the cellulose chains.

Evaluated self-diffusion coefficients along each direction are shown in Table 3 along with an experimental value in bulk water [33], which seemingly supports the above observation. Diffusion of water molecules in confined space has been intensively investigated and it is generally reported that the diffusion coefficient is largely reduced in this kind of gap; see [34] for a review. Surface wettability and atomic-scale structure affect the diffusion [35,36], which explains our results. In the vicinity of hydrophilic surface of cellulose (Type B), water molecules are hydrogen-bonded to cellulose weakly but the diffusion by jumping or hopping is not hindered very much, especially in the direction of crossing cellulose chains (along the *y*-axis). The situation is different on the oleophilic surface (Type C), where hydrogen bonds among water molecules should be enhanced on the hydrophobic pyranose rings, slowing the molecular diffusion.

### 3.6. Interface Deformation

**Figure 9:**
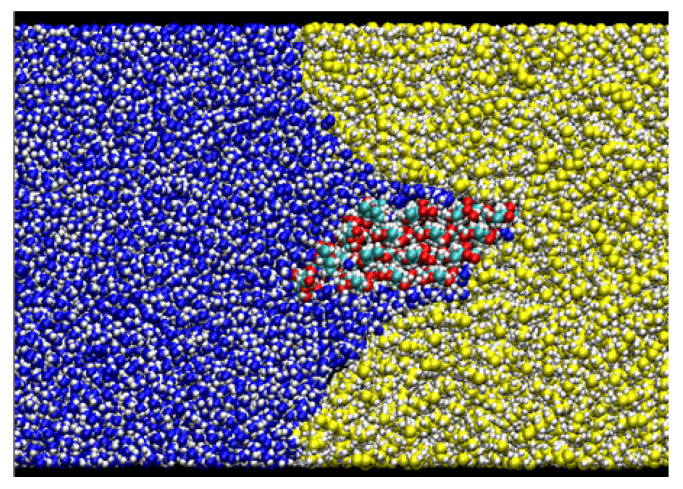
An example of largely deformed water–oil interface; cross-sectional snapshot for $\left(x=10.0\;Å,\theta_{z}=45^{\circ }\right)$ case.

As a result of large hydrophilicity (and oleophobicity) of this cellulose nanocrystal, a large repulsive force is exerted when the crystal approaches the water–oil interface. We observe a large deformation of the interface, as shown in Figure 9, for example. Since the system size along the *y* direction is limited, this deformation prevented us from evaluate the interfacial tension between the water and oil phases directly with the conventional way of using the pressure tensor components.

## 4. Conclusions

To investigate how “amphiphilic” cellulose nanocrystal is adsorbed on the water–oil interface, we performed a series of molecular dynamics simulations at room temperature with water, octane, and a nanocrystal consisting of 4×4 molecular chains of cellulose. By utilizing the system symmetry, we restricted the degrees of freedom of the nanocrystal to the center-of-mass position *x* along the normal of the water–oil interface and the orientation angle $\theta_{z}$ around the crystal axis placed parallel to the interface. A smooth free energy surface $F\left(x,\theta_{z}\right)$ was obtained as the potential of mean force by numerically integrating the mean force and the mean torque exerted on the nanocrystal.

The obtained free energy indicates that the sample of cellulose nanocrystal is highly hydrophilic; *F* in bulk oil phase is higher by 318 kcal/mol than that in bulk water. Three states of local free energy minimum were found. The most stable one is the configuration in which the nanocrystal directly contacts with the oil phase at one crystal edge. In the other two configurations, a thin water layer exists between the crystal surface and the oil phase. The water molecules in the thin layer tend to take special orientations to stabilize the adsorbed nanocrystal, and their translational diffusion is much slower than in bulk water.

Although the nanocrystal of pristine cellulose used here seems too hydrophilic to be an effective emulsifier, a number of chemically modified cellulose materials to control the amphiphilicity have been proposed [2,28]. Recently, even “superhydrophobic cellulose” has been fabricated for oil extraction by polymer coating [37]; it was also reported that structural modification with an electrospinning technique is effective in enhancing the oleophilicity (lipophilicity) of fibrous materials [38]. Our method proposed here to evaluate the adsorption free energy map can be utilized to investigate their adsorption properties at micro scales. The temperature effect is also important [9]; it would be interesting to investigate the temperature dependence of the adsorption free energy, which is hardly accessible by experiments.

## Figures and Tables

**Table 1:** Simulation conditions. The exact number of solvent molecules (water and octane) varies in cases since overlapping molecules are removed when the cellulose nanocrystal is inserted in the system.

Cell Size		205.0 Å × 80.0 Å × 41.4 Å
Temperature		300 K
Density	Water	989.0 kg/m^3^
	Octane	759.0 kg/m^3^
Number of atoms in cellulose nanocrystal		2688
Number of molecules	Water	typically 11,000
	Octane	typically 1300
Distance of potential cutoff in real space		10 Å
Accepted relative errors in PPPM		10^−4^
Time step Δt		0.5 fs
Total number of steps for each case		$5\times 10^{6}$ (2.5 ns)

**Table 2:** Summary of three states of the local minima on the adsorption free energy surface.

	Cross-Sectional View	Enlarged View
Type A: Direct contact $\left(-18\;Å,95^{\circ }\right)$ ΔF=−3.586 kcal/mol	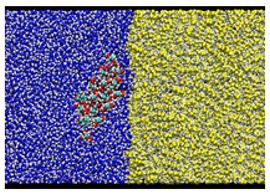	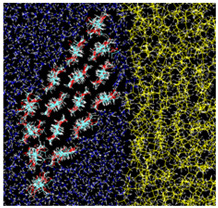
Type B: Hydrophilic surface/Water/Oil $\left(-21\;Å,85^{\circ }\right)$ ΔF=−3.460 kcal/mol	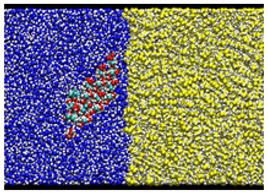	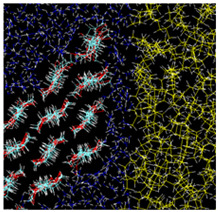
Type C: Oleophilic surface/Water/Oil $\left(-17\;Å,135^{\circ }\right)$ ΔF=−2.384 kcal/mol	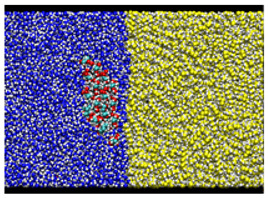	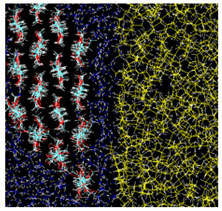

**Table 3:** Diffusion coefficient of water molecules in the gap region between the cellulose nanocrystal and the oil phase.

	In Gap		Bulk	
[10^−8^ m^2^/s]	Type B	Type C	Our Simulation	Experiment [33]
Normal to surface: *D_x_*	<0.02	<0.005		
Crossing chain array: *D_y_*	0.17	0.078	0.27	0.257 (at 25 °C)
Along chains: *D_z_*	0.12	0.085		

## Data Availability

The related data are available on request from the corresponding author.
